# Analyses of Essential Elements and Heavy Metals by Using ICP-MS in Maternal Breast Milk from Şanlıurfa, Turkey

**DOI:** 10.1155/2018/1784073

**Published:** 2018-04-04

**Authors:** Serap Kılıç Altun, Hikmet Dinç, Füsun Karaçal Temamoğulları, Nilgün Paksoy

**Affiliations:** ^1^Department of Food Hygiene and Technology, Faculty of Veterinary Medicine, Harran University, 63200 Şanlıurfa, Turkey; ^2^Department of Pharmacology and Toxicology, Faculty of Veterinary Medicine, Harran University, 63200 Şanlıurfa, Turkey; ^3^Department of Biochemistry, Faculty of Veterinary Medicine, Harran University, 63200 Şanlıurfa, Turkey

## Abstract

Maternal breast milk is a unique biological matrix that contains essential micronutrients. Potentially heavy metals may also affect infants' health and growth through maternal breast milk. The purpose of this study was to determine and compare the essential elements and heavy metals of maternal breast milk of nursery mothers residing in Şanlıurfa province, Turkey. Maternal breast milk concentrations of sodium, magnesium, phosphorus, potassium, calcium, iron, copper, zinc, arsenic, and lead were analyzed in a random sample of the first time in urban and suburban nursery Turkish mothers (*n*: 42). Eight essential elements and two heavy metals were analyzed using ICP-MS after microwave digestion. For bivariate analyses of variables, we use nonparametric Spearman's correlation coefficient test. The mean concentrations of essential elements and heavy metals were as follows: sodium 330 ± 417 mg/L, magnesium 32.6 ± 15.5 mg/L, phosphorus 156 ± 46.2 mg/L, potassium 488 ± 146 mg/L, calcium 193 ± 53.2 mg/L, iron 1.65 ± 1.43 mg/L, copper 0.54 ± 0.46 mg/L, zinc 2.89 ± 3.23 mg/L, arsenic < 1 *μ*g/L, and lead < 1 *μ*g/L. Concentrations of heavy metals in maternal breast milk may have the important implication that it is not affected by environmental pollution in this province. This study provides reliable information about maternal breast milk concentrations of nursery mothers residing in Şanlıurfa, Turkey, and also compares the relations between essential elements and socioeconomic conditions, residing areas, and using copper equipment for food preparation of which some have not previously been reported.

## 1. Introduction

Maternal breast milk (MBM) is a unique biological matrix of nutrition for infants. Breastfeeding is vital for optimal health, growth, and development with its macro- and micronutrients, including essential elements [1]. World Health Organization (WHO) declared that MBM provides ideal nutrition for the first six months of early life [2].

Essential elements in MBM are crucial for ideal growth and development of infants. There are a lot of benefits associated with MBM, such as decreasing the morbidity, mortality, and incidences of diseases [3]. The composition of MBM can be affected by various factors such as maternal diet, social-economic conditions, timing of delivery, period of lactation, and sampling time of day [4]. Essential element deficiencies are correlated with increased rates of infections and chronic diseases. Also, high amount of essential elements may also be harmful to human life [1]. Elements levels depend on host susceptibility which contains physiological conditions, genetic characteristics, age, and gender. Also, lactation is a period of life with increased risks for heavy metals [5] such as arsenic and lead. The concentrations of heavy metals may vary widely depending on exposures. The most common source of lead is the exhaust gases of vehicles [6, 7]. Researchers have shown that lead can pass by maternal way to fetus and by milk to infants. Arsenic is a heavy metal naturally found in the earth and taken up to metabolism by contaminated water and plants. Prolonged exposure to arsenic in the infants can lead to arsenic poisoning and cancer risk [6, 8].

Concentration data on some essential elements and heavy metals in MBM of nursery mothers from different countries analyzed with different techniques such as flame photometry [9], ion chromatography [10], proton-induced X-ray emission, and instrumental neutron activation [11] have been reported. ICP-MS has been used to determine the level of essential elements and heavy metals in various matrices including biological samples and foods [12]. There is, however, no recent study of essential elements and heavy metals in MBM, particularly not from Turkish mothers.

The aim of this study is to provide and access the information of concentrations of essential elements in MBM from nursery mothers who are residing in Şanlıurfa province of Turkey using a sensitive method and also compare the relations between essential elements and socioeconomic conditions, residing areas, and using copper equipment for food preparation.

## 2. Materials and Methods

### 2.1. Breast Milk Sampling

Samples were collected from 42 nursery mothers with an average age of 28.3 ± 7.08 years delivered at full term (38–40 weeks) of gestation. All samples were obtained from nursery mothers' 3–520 lactation days postpartum during the hours of 08:00–11:30 a.m. MBM samples were manually expressed from the breast using a manual pump to sterile falcon tube after 3 mL was discarded and stored at −20°C until analyses. Based on self-reporting on the socioeconomic levels, residing areas, and using copper equipment for food preparation, participants were divided into groups (Table 1).

### 2.2. Chemical and Standard Solutions

Mixed calibration standards of sodium, magnesium, phosphorus, potassium, calcium, iron, copper, zinc, arsenic, and lead were prepared from dilutions of stock solutions (Agilent Japan (Lot Number: 10-160YPY2)). TraceSELECT grade of nitric acid (65.0% concentrated, Merck, Germany) and hydrogen peroxide (30.0% concentrated, Merck, Germany) were used for sample digestion.

Ultrapure water was provided in the laboratory (18.2 MΩ/cm at 25°C) by MES MP Mini pure, (Turkey) device. Every plastic and glassware were cleaned in acid bath (5% nitric acid (laboratory grade, Merck, Germany)) for 24 h and rinsed with ultrapure water and dried before use. The gas 99.9990% Argon was supplied by Linde Gases (Linde Group, Turkey). Whole solutions were prepared with ultrapure water.

### 2.3. Equipment and Accessories

Elements were analyzed on Agilent 7500 ce with an octopole reaction system inductively coupled plasma-mass spectrometer (Agilent Technologies, Japan) with an autosampler (Cetac ASX-520) and a nebulizer (Agilent Technologies, Japan).

### 2.4. Sample Preparation

MBM samples were analyzed by ICP-MS after microwave-assisted acid digestion. 1.0 mL of each sample and blank samples was digested with 4.0 mL of 65% (v/v) HNO_3_, and 2.0 mL of 30% (v/v) H_2_O_2_ in PTFE vessels. Microwave acid digestion procedure was as follows: firstly up to 120°C for 15 min, for the second step constant for 10 min; up to 160°C in 20 min; and finally constant for 15 min; at the end a cooling stage (30 min) was done in room temperature and the samples and blanks were diluted to 50 mL with ultrapure water.

### 2.5. Isotopes

The isotopes ^23^Na^+^, ^24^Mg^+^, ^26^Mg^+^, ^31^P^+^, ^39 ^K^+^, ^44^Ca^+^, ^56^Fe^+^, ^63^Cu^+^, ^66^Zn^+^, ^75^As^+^, and ^208^Pb^+^ were detected. All samples were analyzed in triplicate. These isotopes were preferred to maximize the sensitivity and minimize interferences.

### 2.6. Quality Control

Limit of quantification (LOQ) and limit of detection (LOD) were calculated for ten times and the recovery of 12 elements (Na, Mg, P, K, Ca, Fe, Cu, Zn, As, and Pb) in raw milk samples was between 92.4 and 124.1% as shown in Table 2. The standard deviation of the blank was relative to the slope of the analytical curve. LOQ and LOD were calculated separately. The digested MBM was used in the calculation of LOQ and LOD.

### 2.7. Recovery Experiments

With a view to impose the integrity of the analyses, the same concentrations of each element as in the original sample were added to a sample and performed in the same microwave digestion procedure as the samples.

### 2.8. Statistical Analyses

Statistical analyses were carried out using SPSS 11.00 (SPSS Inc., Chicago, IL, USA). For bivariate analyses of variables, nonparametric Spearman's correlation coefficient was used.

### 2.9. Ethical Committee

Ethics committee approval was obtained from Medical Faculty of Harran University (74059997.050.01.04/65). Informed consents were obtained and signed by the participating nursery mothers to protect the participants' privacy, personal and clinical data, and MBM samples used only for this study purpose.

## 3. Results and Discussion

The nursery mothers included in this study were on the average age of 17–44 (28.3 ± 7.08) years at the time of lactation period average of 3–520 (163 ± 139) day.  Table 3 shows the element levels in MBM samples. Na, Mg, P, K, Ca, Fe, Cu, and Zn were present in all MBM samples (positive rate 100.0%). As and Pb were not present in all MBM samples (negative rate 100.0%). K is the major essential element (488 ± 147 mg/L), followed by Na (330 ± 417 mg/L) > Ca (193 ± 53.2 mg/L) > P (156 ± 46.3 mg/L) > Mg (32.6 ± 15.5 mg/L) > Zn (2.89 ± 3.23 mg/L) > Fe (1.65 ± 1.43 mg/L) > Cu (0.54 ± 0.46 mg/L). The levels of heavy metals arsenic and lead were found below the limit of detection, which is also expected in MBM.

Sodium levels in MBM samples ranged between 44.7 and 1703 mg/L and significantly correlated with copper (*r*: 0.719; *P* < 0.001), iron (*r*: 0.516; *P* < 0.001), and magnesium concentrations (*r*: 0.558; *P* < 0.001). Magnesium concentrations in MBM were in a range of 12.7–85.9 mg/L with a mean level of 32.6 ± 15.5 mg/L. Magnesium and zinc levels were significantly correlated (*r*: 0.474; *P* < 0.01).

MBM phosphorus concentrations were between the level of 84–300 mg/L, with a mean level of 156 ± 46.3 mg/L. Phosphorus concentrations were negatively correlated with iron concentrations (*r*: −0.414; *P* < 0.01), lactation period (*r*: −0.436; *P* < 0.01), and positively correlated with nursery mothers' age (*r*: 0.434, *P* < 0.01).

Potassium concentrations of MBM varied between 161 and 903 mg/L. The content of potassium was positively correlated with zinc (*r*: 0.715; *P* < 0.01), sodium (*r*: 0.519; *P* < 0.01), magnesium (*r*: 0.451; *P* < 0.01), and copper (*r*: 0.719; *P* < 0.001).

Calcium concentrations of MBM were in the range of 90–276 mg/L with a mean level of 193 ± 53.2 mg/L. Calcium was positively correlated with the residing area (*r*: −0.315, *P* < 0.05). Also, calcium levels were significantly correlated with magnesium (*r*: 0.420; *P* < 0.01) and potassium (*r*: 0.525; *P* < 0.01). Concentrations of calcium in MBM from suburban nursery mothers were significantly lower than concentrations of calcium in urban nursery mothers.

The mean concentration of iron in MBM was 1.65 ± 1.43 mg/L. Copper concentration of MBM was positively correlated with sodium (*r*: 0.719; *P* < 0.001), magnesium (*r*: 0.673; *P* < 0.001), and lactation period (*r*: 0.832; *P* < 0.001) and with using copper equipment in food preparation (*r*: 0.333; *P* < 0.05).

The zinc concentrations were positively correlated with sodium (*r*: 0.586; *P* < 0.001) and potassium (*r*: 0.715; *P* < 0.001) concentrations of MBM samples.

In this study, the results showed the differences in essential elements and heavy metals concentrations in MBM of nursery mothers residing in Şanlıurfa, Turkey. Specifically, nursery mothers from the suburban area (Eyyübiye) of Şanlıurfa tended to have lower intake concentrations of calcium assessed as compared with nursery mothers residing in urban areas (Haliliye and Karaköprü). As a result, MBM from nursery mothers residing in suburban area (Eyyübiye) were of poorer nutritional quality that has obvious implication for infants nutrition. Arising from this result, family population was crowded and socioeconomic levels of these families were low. The results of this study were parallel with Qian et al.s' [10] study who determined and compared the composition of MBM in urban and suburban areas in Shanghai, China.

Calcium is the principal element of skeleton system that improves bone mineral density in infants. Our findings show that maternal calcium intake was lower in suburban nursery mothers than other participants of this study. Qian et al. [10] reported that maternal calcium intake was low in both urban and suburban groups of nursery mothers. The concentrations of in MBM ranged from 90 to 276 mg/L in this study. In Sweden, Björklund et al. [12] reported 274–305 mg/L calcium levels in 60 MBM samples. Supposing a daily intake of 750 mL breast milk, infants should obtain 67.5–207 mg of calcium per day. For age 0–6 months, the concentration of calcium is lower daily intake that recommended amount of 210 mg [13].

There is evidence that sodium levels in infancy may have a long-term effect on blood pressure. The observation was that mean concentrations of sodium were higher than the previous studies [9, 14]. Mean sodium concentration of our study was 330 ± 417 mg/L. Our sodium concentrations were similar to MBM samples of the urban area of Shanghai but higher than the MBM samples from the suburban area of Shanghai [10]. And also our mean level of sodium was higher than the mean level of sodium (135 mg/L) from Japanese mothers' [15] and the rural Gambian mothers' MBM with a mean level of sodium (163 mg/L) [9]. The high sodium concentrations of this study may be due to dietary habits of Turkish women with a consumption of salt 14 g/day [16].

MBM samples seem to contain enough concentration of magnesium as there has not been a report of magnesium deficiency in breastfed infants [11]. The levels of magnesium in MBM samples were in the range of 12.7–85.9 mg/L with a mean level of 32.6 ± 15.5 mg/L which was in accordance with Yamawaki et al.s' study [15] (mean 27 ± 9 mg/L). Magnesium concentrations in MBM samples were with values similar to Swedish mothers with an average level of magnesium 28 mg/L [12].

We also found that phosphorus concentrations were in a negative correlation with lactation period. The measured phosphorus concentrations in MBM samples ranged within 84–300 mg/L with an average level of 156 ± 46.3 mg/L which was similar to Japan nursery mothers P levels with an average level of 150 ± 38 mg/L [15], Shanghai nursery mothers with a range of 139–163 mg/L phosphorus levels [8], and Swedish mothers with a range of 126–233 mg/L [12].

Potassium is an intracellular cation [11]. Average concentrations of potassium varied between 161 and 903 mg/L with a mean level of 488 ± 147 mg/L. Comparing our data with those of other studies in which MBM samples were gathered during lactation in, for example, Shanghai [10], Japan [15], and Sweden [12] shows similarity in concentrations of potassium.

There have been few reports about iron content in MBM [5, 15, 17]. Iron is an essential element for ideal growth under the case of oxidative stress [5]. Our mean data of iron was 1.65 ± 1.43 mg/L with levels that confirm the results reported in the literature [5, 18]. The concentration of iron was found to be higher than the Uppsala mothers with median level of 0.31 mg/L [9], and American mothers living in South Texas with a mean level of 0.4 mg/L [4], and lower than in Croatian nursery mothers with a mean level of 3.3 mg/L [5].

The mission of essential elements in biological functions makes them especially significant for nutrition of infant. It is noted that an infant is well protected so long as certain age by maternal homeostatic processes in the event of iron, copper, and zinc [19]. Infants are born with an adequate supply of iron and copper in the liver, owing to low concentrations of these elements situated in MBM [20].

Zinc and copper are essential elements which support development and represent significant levels in MBM [21]. We found that zinc levels were in the range of 0.45–15.8 mg/L with a mean level of 2.89 ± 3.23 mg/L. Assuming a daily intake of 750 mL, infants should obtain 2.16 mg of zinc per day. This concentration is suitable daily intake for age of 0–6 months that is recommended [22]. Zinc concentrations in MBM are regulated by the mammary glands [23]. Reported average concentration of zinc in MBM was between 1.24 and 20 mg/L [5, 10, 12, 17, 19].

It is presumed that the transition of copper from blood to milk regulated transfer by the mammary gland epithelium [19]. Consequently, there was not any correlation between the concentrations of copper in mother's blood and milk which was expected [19, 24]. The levels of copper in MBM in Croatia were 3.4 ± 0.7 mg/L. Our results of copper concentrations with a mean 0.54 ± 0.46 mg/L are lower than the reported study [5]. Mohd-Taufek et al. [18] reported a mean concentration of copper 0.22 ± 0.03 mg/L and also Yamawaki et al. reported [15] similar copper concentrations with a mean level of 0.35 ± 0.21 mg/L. The results of the present study (range 0.08–2.01 mg/L) were very similar to those found by Almeida et al. [19].

By detecting the concentrations of heavy metals (arsenic and lead) of MBM, we estimated that no influences from the maternal intake or environmental exposure to these elements by reason of the concentrations were below the detection limit. This result may be because the Şanlıurfa province is away from the industry. Compared with concentrations reported in the literature [5, 19] the arsenic and lead levels detected in this study were so much lower than most of the reported values.

We should be noted that MBM samples were obtained in one-time collection. Next research needed the composition of MBM varying on a time to time basis. Serial analyses of MBM samples in repeated times should address the concentrations of essential elements and heavy metals of Turkish mothers.

## 4. Conclusion

In brief, our results agree on the whole with previous reports about the essential element composition of maternal breast milk but it was clearly understood that there is a large standard variation in the composition of essential elements such as Ca, Fe, and Zn. We confirmed that the composition of human milk is affected by various factors such as residing area and using copper equipment.

In conclusion, we found a significant correlation in MBM concentrations between nursery mothers from urban and suburban areas of Şanlıurfa. More researches at multiple-time compositional studies are warranted to detect the concentrations for identifying appropriate interventions to ensure healthy growth and development of breastfed infants.

## Figures and Tables

**Table 1:** Data of nursery mother and the infants' status.

Parameter	Total number (*n*)	Percentage (%)	Min	Max	Mean ± SD
Age of infant (day)	42	100.0	3	520	162 ± 139
Age of mother (year)	42	100.0	17	44	28.3 ± 7.08
Income rate < minimum wage	21	50.0			
Income rate ≥ minimum wage	21	50.0			
Residing in urban area	25	59.5			
Residing in suburban area	17	40.4			
Using copper equipment	13	31.0			
Not using copper equipment	29	69.0			

**Table 2:** Quantification and detection limits.

Element	LOQ	LOD	Concentration range of standard solution
Na (mg/L)	315	10.0	1–2000
Mg (mg/L)	48.6	4.60	1–100
P (mg/L)	562	35.0	10–1000
K (mg/L)	1413	100	10–1000
Ca (mg/L)	678	22.0	10–1000
Fe (mg/L)	2.53	0.40	0.1–1000
Cu (mg/L)	0.06	0.01	0.01–100
Zn (mg/L)	1.81	0.20	0.1–100
As (*µ*g/L)	0.04	0.01	0.1–100
Pb (*µ*g/L)	0.04	0.01	0.1–100

**Table 3:** Concentration of essential elements and heavy metals of MBM samples.

	Min	Max	Mean ± SD
*Essential elements*			
Na (mg/L)	44.7	1703	330 ± 417
Mg (mg/L)	12.7	85.9	32.6 ± 15.5
P (mg/L)	84.0	300	156 ± 46.3
K (mg/L)	161	903	488 ± 147
Ca (mg/L)	90.0	276	193 ± 53.2
Fe (mg/L)	0.45	5.11	1.65 ± 1.43
Cu (mg/L)	0.08	2.02	0.54 ± 0.46
Zn (mg/L)	0.45	15.8	2.89 ± 3.23
*Heavy metals*			
As (*µ*g/L)	<1	<1	<1
Pb (*µ*g/L)	<1	<1	<1
